# Mitochondrial Genome Analyses Suggest Multiple *Trichuris* Species in Humans, Baboons, and Pigs from Different Geographical Regions

**DOI:** 10.1371/journal.pntd.0004059

**Published:** 2015-09-14

**Authors:** Mohamed B. F. Hawash, Lee O. Andersen, Robin B. Gasser, Christen Rune Stensvold, Peter Nejsum

**Affiliations:** 1 Department of Veterinary Disease Biology, University of Copenhagen, Frederiksberg, Denmark; 2 Zoology Department, Faculty of Science, Cairo University, Giza, Egypt; 3 Department of Microbiology and Infection Control, Statens Serum Institut, Copenhagen, Denmark; 4 Department of Veterinary and Agricultural Sciences, University of Melbourne, Parkville, Victoria, Australia; University of Pennsylvania, UNITED STATES

## Abstract

**Background:**

The whipworms *Trichuris trichiura* and *Trichuris suis* are two parasitic nematodes of humans and pigs, respectively. Although whipworms in human and non-human primates historically have been referred to as *T*. *trichiura*, recent reports suggest that several *Trichuris* spp. are found in primates.

**Methods and Findings:**

We sequenced and annotated complete mitochondrial genomes of *Trichuris* recovered from a human in Uganda, an olive baboon in the US, a hamadryas baboon in Denmark, and two pigs from Denmark and Uganda. Comparative analyses using other published mitochondrial genomes of *Trichuris* recovered from a human and a porcine host in China and from a françois’ leaf-monkey (China) were performed, including phylogenetic analyses and pairwise genetic and amino acid distances. Genetic and protein distances between human *Trichuris* in Uganda and China were high (~19% and 15%, respectively) suggesting that they represented different species. *Trichuris* from the olive baboon in US was genetically related to human *Trichuris* in China, while the other from the hamadryas baboon in Denmark was nearly identical to human *Trichuris* from Uganda. Baboon-derived *Trichuris* was genetically distinct from *Trichuris* from françois’ leaf monkey, suggesting multiple whipworm species circulating among non-human primates. The genetic and protein distances between pig *Trichuris* from Denmark and other regions were roughly 9% and 6%, respectively, while Chinese and Ugandan whipworms were more closely related.

**Conclusion and Significance:**

Our results indicate that *Trichuris* species infecting humans and pigs are phylogenetically distinct across geographical regions, which might have important implications for the implementation of suitable and effective control strategies in different regions. Moreover, we provide support for the hypothesis that *Trichuris* infecting primates represents a complex of cryptic species with some species being able to infect both humans and non-human primates.

## Author Summary

*Trichuris trichiura* and *Trichuris suis* are whipworms found in humans and pigs, respectively, causing morbidity in humans and being associated with production losses in pigs. Although *Trichuris* from non-human primates is attributed to *T*. *trichiura*, hence considered the same species as the one infecting humans, several recent reports question this assumption. Morphologically similar parasites that have a wide global distribution and/or those capable of infecting several host species may comprise several ‘hidden’ species. In this study, we sequenced, annotated, and compared the mitochondrial genomes (including published genomes) of *Trichuris* obtained from different hosts in different geographical regions, including humans (Uganda and China), pigs (China, Uganda, and Denmark) and two types of non-human primates (baboons and françois’ leaf monkey). We found high genetic distinctiveness between human *Trichuris* from China and Uganda. Likewise, pig *Trichuris* from Denmark and other regions also showed considerable, although lower, genetic diversity. This suggests that both pig- and human-derived *Trichuris* may represent different species with potential differences in endemicity, which may have important implications for implementing effective control strategies. Our data also suggests that *Trichuris* infecting primates comprises several species and may be transmitted from non-human primates to humans.

## Introduction

Neglected tropical diseases, including helminthiases, have a devastating effect on human health. It is estimated that about one billion people are infected with soil-transmitted helminths (STHs), including the common roundworm (*Ascaris*), hookworms (*Necator* and *Ancylostoma* spp.), and whipworm (*Trichuris*), mostly in underprivileged regions of the world [1]. Approximately 0.5 billion people are infected with *T*. *trichiura*, resulting in the loss of 0.64 million disability-adjusted life years [2]. Compared with adults, children are more prone to developing clinical symptoms such as dysentery, bloody diarrhea, rectal prolapse, and cognitive impairment in cases of chronic infection [3, 4].

Although whipworm infections in non-human primates are usually called *T*. *trichiura*, recent studies suggest that primates may host multiple species of *Trichuris*. Ravasi et al. [5] found evidence of two *Trichuris* species in both baboons and humans based on the sequences of the internal transcribed spacers (ITS) of nuclear ribosomal DNA. Another study by Hansen et al. [6] based on studies of the beta-tubulin gene and ITS-2 sequencing suggested that humans and baboons host shared *Trichuris* species. On the other hand, Liu et al. [7] identified a potentially novel species of *Trichuris* in a non-human primate (françois’ leaf monkey) based on complete mitochondrial genome analysis and the ITS-1 and -2 regions. Recently, Ghai et al. [8] suggested that *Trichuris* spp. in human and non-human primates represent several species that differ in host specificity. Therefore, there is a need to further explore which species of *Trichuris* that infect primates and investigate potential (zoonotic) routes of transmission between host species.

The whipworm of pigs, *Trichuris suis* is associated with production losses due to reduced growth rates and lower feed conversion efficiency [9]. Although morphologically indistinguishable from *T*. *trichiura*, several studies identified extensive genetic diversity between *T*. *trichiura* and *T*. *suis* based on nuclear and mitochondrial DNA analysis [10–12]. However, molecular characterization of *Trichuris* from sympatric pigs and humans indicated that *T*. *suis* can cause zoonotic infection in humans, emphasizing the public health importance of this pig parasite [12].

The circular mitochondrial (mt) genomes are relatively small in size (13–26 kb) and encode enzymes required for oxidative phosphorylation. Mitochondrial DNA has a number of advantages for delimiting closely related species due to its high substitution rate coupled with its low effective population size, which leads to rapid lineage sorting following speciation [13]. Comparative mitochondrial DNA analysis is therefore useful for identifying cryptic (“hidden”) species, i.e., those that cannot be differentiated by traditional methods, including morphological analysis. On the other hand, mitochondrial pseudogenes (numts) in the nuclear genome may lead to incorrect phylogenetic inferences, which is why caution is warranted whenever mt genes are used in phylogenetic analyses [14]. Moreover, sole dependence on mtDNA for delineating the taxonomic status might also lead to ambiguous phylogeny and misidentification of individuals due to incomplete lineage sorting or mitochondrial introgression [15].

Parasites with a wide geographical distribution or multiple host species may comprise cryptic species [13]. For instance, *Hypodontus macropi*, an intestinal parasite in macropodid marsupials, was found to consist of several cryptic species based on mt genome analysis [16]. In the study by Blouin [17], the genetic difference between sibling nematode species typically ranges between 10%–20% using *cox*1 and *nad*4 mt genes, whereas intra-species variation is usually below 2%.

In the present study, we logically extend previous investigations to investigate levels of genetic variation among specimens of *Trichuris* from a human from Uganda, two baboons and pigs from Denmark and Uganda. To do this, we (i) sequenced and characterized complete mt genomes from individual adult worms from these three host species and (ii) compared them (at the amino acid sequence level) with those of *Trichuris* spp. of human, françois’ leaf-monkey and pig determined in previous studies, in order to assess levels of genetic variation within *Trichuris* among host species and geographical regions.

## Methods

### Ethics statement

The human *Trichuris* was recovered from the feces of a child after anthelmintic treatment as part of an efficacy study as described previously [12]. Permission was obtained from the Ministry of Health and the National Council of Science and Technology in Uganda, and the Danish Central Medical Ethics Committee approved the study. The parents and children were informed about the study and received a consent form in both English and the local language. Written informed consent was received for each individual participating in the study. Worms from baboons in the Southwest National Primate Research Center, Texas, USA and the Copenhagen Zoo, Denmark were recovered during post mortem examination, which is performed both places on all animals culled on a routine basis. *T*. *suis* was obtained from an experimentally infected pig in Denmark. The Animal Experiments Inspectorate, Ministry of Justice, Denmark, approved the animal study protocol, which was carried out according to stipulated guidelines (License no. 2005/561-1060). *T*. *suis* was obtained from a naturally infected pig in Uganda raised on a private farm, slaughtered, and used for local consumption. Permission to recover worms from the animal was obtained from the owner.

### Parasites, DNA extraction, and genotyping of worms

Adult *Trichuris* worms were recovered from an olive baboon, *Papio anubis*, at Southwest National Primate Research Center, Texas, USA, and a hamadryas baboon, *Papio hamadryas*, in Copenhagen Zoo, Denmark, both during post mortem examination. Adult *Trichuris* were collected from domesticated pigs post mortem from Denmark and Uganda and recovered from a human stool sample from Uganda upon anthelmintic treatment as described [12]. Worms were rinsed with tap water and stored in 70% ethanol at 5°C until DNA extraction.

The MasterPure DNA Purification Kit (Epicenter Biotechnologies) was used to extract total genomic DNA from the anterior thin part of the worms according to manufacturer's protocol. Worm material was homogenized in lysis solution in an Eppendorf tube using a matching plastic pestle followed by incubation at 56°C for at least six hours.

PCR-linked restriction fragment length polymorphism analysis (PCR-RFLP) of the internal transcribed spacer-2 (ITS-2) region was used to genotype worms, since *Trichuris* from primates, including humans, and pigs are morphologically indistinguishable [8, 12]. PCR products and digested fragments were resolved using 1.5% agarose gels, stained with GelRed^TM^ (Biotium), and detected using UV light. Worms from humans and baboons showed banding patterns characteristic of *T*. *trichiura* (~130, 220 and 340 bp) and worms from pigs showed banding patterns characteristic of *T*. *suis* (~130 and 490 bp) [12].

### Mitochondrial genome amplification and sequencing

Different primate- and pig-derived *Trichuris* were chosen for long-range PCR amplification and next generation sequencing (NGS). Two baboon worms, *P*. *hamadryas* (TTB1) and *P*. *anubis* (TTB2), one Uganda human worm (TTHUG), and two pig worms (TSDK and TSUG) from Denmark and Uganda, respectively were chosen based on their distinct haplotypes identified as part of another study when sequencing the *rrn*L gene of 140 *Trichuris* worms. The two mt genomes of *T*. *trichiura* and *T*. *suis* (Accession nos. GU385218 and GU070737, respectively) were aligned to identify conserved regions relevant to primer design. However, no suitable conserved regions were identified, which precluded the design of general primers applicable to all worm samples. Hence, different sets of primers were designed for each genome. Primers were designed based on the genome of *T*. *trichiura* (GU385218) to amplify the mt genomes of the baboon- and human-derived *Trichuris* in three overlapping fragments (~5 kbp each) and for pig-derived *Trichuris* TSDK and TSUG in three overlapping fragments (~6, 5, and 3 kbp) based on the genome of *T*. *suis* (GU070737) (Table 1). However, several obstacles were encountered in the amplification and sequencing processes. First, only TTB2 was amplified, and other sets of primers were therefore designed to amplify the TTB1 and TTHUG genomes in two overlapping fragments (~8 and ~6 kbp) (Table 1). However, due to the presence of non-specific bands, amplified DNA from the band representing the fragment *nad*1–*rrn*L was extracted from the agarose gel using spin columns (Millipore) as stipulated by the manufacturer’s protocol. Second, the library construction (see below) of the TTHUG genome failed, and the genome was amplified in 15 fragments of ~1,000 bp each, using 15 overlapping primer pairs designed based on the TTB1 mt genome (S1 Table) and sequenced by Sanger dideoxy-sequencing (Macrogen Inc., Seoul, South Korea).

**Table 1:** Primers used for complete mitochondrial genome amplification of *Trichuris* from baboons (TTB1 and TTB2) and pigs (TSDK and TSUG).

	Forward (5’—3’)		Reverse (5’—3’)
**TTB2**			
**TTB2*cox*1F**	CAGGAAATCACAAGAAAATTGG	**TTB2*nad*5R**	AGTGGTTGCAGGAACAATTC
**TTB2*nad*5F**	AGCAATCTGCGATATTGTTG	**TTB2*rrn*LR**	TCGCAACGGTTTAAACTCAA
**TTB2*rrn*LF**	CGCAGTAATCTGACTGTGC	**TTB2*cox*1R**	AAATTTTCCTGCTATGAATATGA
**TTB1**			
**TTB1*nad*1F**	ACAGCCCATCCTAGACGGTA	**TTB1*rrn*L**	ACCTGTCTCGCAACGGTTTA
**TTB1*rrn*LF**	TCTGACTGTGCAAAGGTAGCA	**TTB1*nad*1R**	TTGCGGACCAAAAGGTTATGAAT
**TSDK & TSUG**			
**TS*rrn*LF**	TTAAATGGCCGCAGTAACCT	**TS*nad*1R**	AGCTCACCCTGTAATAATGATGT
**TS*nad*1F**	TCTGATCTGTGCTACCCTACAC	**TS*nad*5R**	CCAACACCCGTGAGTTCTT
**TS*nad*5F**	CTTTTGCAAGGGCATGATTA	**TS*rrn*LR**	TCACGTAATGTAGAATCGTCGA

Long-range PCR was conducted in a total volume of 20 μL containing 2 μL 10X PCR buffer, 0.2 mM of each dNTP, 0.4 mM of each primer pair, 2.0 mM MgCl2, and 2.5 U of Long PCR Enzyme Mix (Thermo Scientific). PCR cycling conditions included initial denaturation at 92°C for 4 min, followed by 35 cycles of denaturation at 92°C (20 s), annealing at 50°C (30 s), extension at 62–67°C (7 min), and a final extension at 60–67°C for 10 min. PCR gradient and MgCl_2_ titration was used to optimize the PCR for each primer pair. PCR products were stained using GelRed^TM^ (Biotium) and visualized after gel electrophoresis (0.8% agarose) under UV light. PCR products were cleaned enzymatically using 1 μL Exonuclease I (Fermentas) and 2 μL FastAP Thermosensitive Alkaline Phosphatase (1 U/μL) (Fermentas) for each 5μL of amplicons and incubated for 15 min at 37°C, followed by 15 min at 85°C. Finally, DNA concentration was measured using a NanoDrop 1000 spectrophotometer (Thermo Fischer Scientific), and equal amounts of fragments of each genome were pooled. Library construction, including tagging (indexing) of samples and NGS using the Illumina HiSeq 2000 platform, was performed by Macrogen Inc. (Seoul, South Korea).

### Assembly, annotation, and genome sequence analysis

Reads (~100 bp) of each genome were assembled using CLC Genomics Workbench v.6.0.4 (CLC Inc, Aarhus, Denmark) *de novo* except for sample TSDK that was assembled using TSUG and GenBank entry GU070737 (TSCH). The files of the NGS raw data can be provided upon request. For TTHUG, sequences were manually checked, edited, and trimmed using Vector NTI [18] and BioEdit [19] and aligned to TTB1. After assembly, genome annotation was performed using the pipeline MITOS [20] and BLAST search tools available through NCBI (http://blast.ncbi.nlm.nih.gov/Blast.cgi). Secondary structures for all tRNAs were predicted using tRNAScan-SE [21] and ARWEN [22].

The genomes were compared with *T*. *trichiura* from a human in China (TTHCH) (GU385218); *Trichuris* sp. GHL from francois' leaf monkey (T.GHL) from China (KC461179), and *T*. *suis* from China (TSCH) (GU070737). Protein-coding genes (PCGs) and ribosomal DNA genes were individually extracted and aligned by ClustalW using default settings. Another data set was generated by concatenating all PCG and rDNA sequences. Genetic distances were estimated for these data sets using MEGA v.6.1 [23]. Nucleotide diversity (π) was calculated across the genomes of *Trichuris* from humans and non-human primates and *Trichuris* from pigs using a sliding window of 100 bp with 25 bp steps implemented in DnaSP v.5 [24].

### Phylogenetic analysis

Three different methods were used for phylogenetic analysis, namely Neighbor Joining (NJ), Maximum Likelihood (ML), and Bayesian Inferences (BI). Two different data sets (DNA and amino acid sequences) were generated for the phylogenetic analyses. Amino acid sequences for the 13 PCGs were aligned using ClustalW for 10 *Trichuris* spp., namely *Trichuris* from baboons (TTB1, TTB2), from humans (TTHUG and TTHCH), from francois' leaf monkey (KC461179), from pigs (TSUG, TSDK and TSCH), and from *T*. *ovis* and *T*. *discolor* (JQ996232 and JQ996231, respectively). Similarly, the DNA sequences representing the PCGs and rDNA genes were aligned using ClustalW. *Trichinella spiralis* (AF293969) was used as an outgroup in the phylogenetic analyses. ML and NJ trees were generated using MEGA v.6.1 [23]. The best-to-fit substitution model was identified using jModelTest0.1.1 [25] under Akaike information criterion (AIC) [26] for each dataset. BEAST v.1.6.1 [27] was used for the BI on the two data sets. Uncorrelated log normal was used as prior for the mutation rate with mtRev as the substitution model for protein sequences and the General Time Reversible (GTR) model for DNA sequences, with gamma distribution and invariant sites assumed in both substitution models. A random starting tree with Yule prior was assumed as well. Three independent runs with 10 million steps each with a burn-in of 10,000 steps were carried out. Tracer v.1.6 [27] was used to analyze log files of the MCMC chains, and the reliability of parameters was checked by recording effective sample size values above 200. Tree Annotater v.1.6.1 [27] was used to summarize the tree data with a posterior probability limit of 0.5.

### *Cox*1 phylogeny

In order to investigate the phylogenetic relationship between the mt genome haplotypes identified in this study with other *Trichuris* haplotypes from primates and pigs in different geographical regions, partial (372bp) *cox*1 sequences from GenBank were obtained (Table 2) for phylogenetic analyses. ML and NJ trees were generated using MEGA v.6.1 [23], and the best-to-fit model was identified using jModelTest0.1.1 [25] under Akaik information criterion (AIC) [26]. *Ascaris lumbricoides* (AB591799) was used as an outgroup.

**Table 2:** Partial *cox*1 sequences retrieved from the GenBank database with accession numbers, host, and the country from which the worms were sampled. All the non-human primates represented in the table were held in captivity.

Host	Country	Accession No. in GenBank
***Colobus guereza kikuyensis* (Black-and-white colobus)**	Spain	HE653116, HE653117, HE653118, HE653119.
***Papio anubis* (Olive baboon)**	Czech Republic	JF690964
***Theropithecus gelada* (Gelada baboon)**	Czech Republic	JF690965
***Papio hamadryas* (Hamadryas baboon)**	Czech Republic	JF690963
***Macaca fascicularis* (Longtailed macaque)**	Czech Republic	JF690967
**Human**	Czech Republic	JF690962
***Sus scrofa domestica* (Domestic pig)**	China	HQ204208, HQ204209, HQ183740, HQ183741
***Sus scrofa domestica* (Domestic pig)**	Spain	HE653124, HE653125, HE653126
***Sus scrofa scrofa* (Wild boar)**	Spain	HE653127, HE653128, HE653129

## Results

### Annotation and features of mitochondrial genomes

The complete mt genomes of the primate worms TTB1, TTB2, and TTHUG comprised 13,984, 14,009, and 14,079 bp, respectively, whereas the two pig worms, TSDK and TSUG comprised 14,521 and 14,410 bp (GenBank accession nos. KT449822-KT449826). The genomes contain 13 PCGs, 22 tRNAs, and two ribosomal RNA genes (Tables 3 and 4). The general mt features, including gene synteny, is the same as previously described for *Trichuris* spp. [7, 11,28] as all genes are transcribed from the heavy strand, except 4 PCGs (*nad*2, *nad*5, *nad*4, and *nad*4L) and 10 tRNA motifs (tRNA-Met, tRNA-Phe, tRNA-His, tRNA-Arg, tRNA-Pro, tRNA-Trp, tRNA-Ile, tRNA-Gly, tRNA-Cys, and tRNA-Tyr), which are transcribed from the light strand. The starting and termination codons for some PCGs differed between *Trichuris* spp. recovered from identical host species. For instance, the starting codon for TSDK is ATA for the *nad*4 gene, while it reads ATG in the TSUG genome; for the *atp*6 gene, the starting codon is GTA in TSDK, while being GTG in TSUG. Moreover, the termination codon in the *cox*1 gene is TAG for TSDK and TAA for TSUG, and in the *nad*4 gene, TAA is the termination codon in TSDK, while it reads TAG in TSUG.

**Table 3:** Mitochondrial genomes of baboon *Trichuris* (TTB1 and TTB2) and human *Trichuris* (TTHUG). Protein coding, transfer RNA (tRNA), and ribosomal DNA (rDNA) genes with lengths in nucleotides (nt) are given. The lengths of TTB1 and TTHUG are identical, and differences are given in parentheses for (TTB2/TTHUG); likewise for the initiation and termination codons.

Genes	Positions			Lengths	Codons		Strand
	TTB2	TTB1	TTHUG	nt	Initiation	Termination	
***cox*1**	1–1545	1–1545	1–1545	1545	ATG	TAA	+
***cox*2**	1560–2234	1558–2232	1558–2232	675	ATG	TAG(TAA)	+
**tRNA-leu**	2248–2308	2255–2317	2255–2317	63(-3)			+
**tRNA-glu**	2318–2374	2324–2384	2324–2384	61(-4)			+
***nad*1**	2397–3296	2406–3305	2406–3305	900	ATA	TAG(TAA)	+
**tRNA-lys**	3424–3484	3334–3399	3425–3490	66(-5)			+
***nad*2**	3487–4383	3397–4293	3488–4384	897(-12)	ATA(GTA)	TAA	-
**tRNA-met**	4384–4444	4294–4354	4385–4445	61			-
**tRNA-phe**	4441–4496	4349–4405	4440–4496	57(-1)			-
***nad*5**	4496–6043	4397–5953	4488–6044	1557(-9)	ATA	TAG(TAA)	-
**tRNA-his**	6041–6094	5947–6004	6038–6095	58(-4)			-
**tRNA-arg**	6096–6158	6006–6069	6097–6160	64			-
***nad*4**	6160–7371	6074–7294	6165–7382	1221(-9/-3)	ATG	TAA	-
***nad*4L**	7402–7650	7317–7529	7405–7617	213(+36)	ATA	TAA	-
**tRNA-thr**	7655–7710	7570–7627	7658–7715	58(-2)			+
**tRNA-pro**	7712–7770	7627–7685	7715–7773	59			-
***nad*6**	7763–8239	7678–8154	7766–8242	477	ATT	TAA	+
***cyt*b**	8246–9352	8161–9267	8249–9355	1107	ATG	TAG	+
**tRNA-ser**	9351–9400	9266–9318	9354–9406	53(-3)			+
***rrn*S**	9393–10086	9311–10009	9399–10102	699(-5)			+
**tRNA-val**	10044–10144	10011–10067	10104–10160	57			+
***rrn*L**	10144–11153	10069–11077	10162–11170	1009(+2)			+
***atp*6**	11124–11963	11048–11860	11141–11953	813(+27)	ATG(GTG)	TAA	+
***cox*3**	11938–12711	11866–12639	11959–12732	774	ATG	TAA	+
**tRNA-trp**	12718–12780	12652–12714	12745–12807	63			-
**tRNA-gln**	12784–12836	12718–12773	12811–12866	56(-3)			+
**tRNA-Ile**	12838–12898	12776–12836	12869–12929	61			-
**tRNA-gly**	12908–12964	12850–12906	12943–12999	57			-
**tRNA-asp**	12970–13034	12913–12970	13006–13063	58(+8)			+
***atp*8**	13016–13180	12959–13126	13052–13219	168(-3)	ATT(ATA)	TAG	+
***nad*3**	13190–13531	13136–13477	13229–13570	342	ATT	TAA	+
**tRNA-ser**	13626–13675	13571–13620	13664–13714	50			+
**tRNA-asn**	13676–13729	13621–13675	13715–13769	55(-1)			+
**tRNA-leu**	13737–13799	13683–13742	13777–13836	60(+3)			+
**tRNA-ala**	13806–13858	13754–13811	13848–13905	58(-4)			+
**tRNA-cys**	13888–13940	13854–13907	13948–14001	54(-1)			-
**tRNA-tyr**	13949–14009	13908–13968	14002–14062	61			-

**Table 4:** Mitochondrial genomes of pig *Trichuris* TSDK and TSUG from Denmark and Uganda, respectively. Protein-coding, transfer RNA (tRNA), and ribosomal DNA (rDNA) genes are indicated with lengths in nucleotides (nt) for the respective genes. Gene lengths are given for TSDK, and the differences from TSUG are given in parentheses; likewise for the initiation and termination codons.

Regions	Positions		Lengths	Codons		Strand
	TSDK	TSUG	nt	Initiation	Termination	
***cox*1**	1–1542	1–1542	1542	ATG	TAG(TAA)	+
***cox*2**	1579–2259	1577–2257	681	ATG	TAA	+
**tRNA-leu**	2271–2330	2269–2328	60			+
**tRNA-glu**	2338–2394	2336–2392	57			+
***nad*1**	2416–3315	2414–3313	900	ATT	TAA	+
**tRNA-lys**	3447–3506	3454–3513	60			+
***nad*2**	3521–4402	3528–4409	882	ATA	TAG	-
**tRNA-met**	4412–4473	4419–4480	62			-
**tRNA-phe**	4477–4537	4484–4542	61(-2)			-
***nad*5**	4528–6084	4533–6089	1557	ATA	TAG	-
**tRNA-his**	6085–6140	6090–6145	56			-
**tRNA-arg**	6144–6207	6149–6212	64			-
***nad*4**	6213–7616	6218–7606	1404(-15)	ATA(ATG)	TAA(TAG)	-
***nad*4L**	7726–7968	7628–7891	240(-21)	ATA	TAG	-
**tRNA-thr**	7970–8024	7884–7938	55			+
**tRNA-pro**	8017–8087	7944–8001	71(-14)			-
***nad*6**	8080–8550	7994–8464	471	ATT	TAA	+
***cyt*b**	8563–9675	8476–9588	1113	ATG	TAG	+
**tRNA-ser**	9674–9728	9587–9641	55			+
***rrn*S**	9726–10435	9639–10349	710			+
**tRNA-val**	10435–10491	10349–10405	57			+
***rrn*L**	10500–11510	10414–11420	1011			+
***atp*6**	11506–12309	11410–12219	804(+6)	ATT(GTG)	TAA	+
***cox*3**	12318–13094	12229–13005	777	ATG	TAA	+
**tRNA-trp**	13099–13165	13010–13076	67			-
**tRNA-gln**	13169–13225	13080–13136	57			+
**tRNA-ile**	13228–13293	13139–13204	66			-
**tRNA-gly**	13312–13367	13223–13278	56			-
**tRNA-asp**	13382–13441	13290–13350	60			+
***atp*8**	13421–13591	13331–13501	171	TTG	TAA	+
***nad*3**	13616–13957	13526–13867	342	ATA	TAA	+
**tRNA-ser**	14065–14118	13975–14026	54(-2)			+
**tRNA-asn**	14118–14177	14026–14084	60(-1)			+
**tRNA-leu**	14193–14252	14102–14164	60(+3)			+
**tRNA-ala**	14258–14312	14167–14221	55			+
**tRNA-cys**	14344–14401	14245–14299	58(-3)			-
**tRNA-tyr**	14401–14457	14300–14356	57			-

Similar observations were found in the *Trichuris* genomes from baboons. For *nad*2 and *atp*6, the starting codons were ATA and ATG, respectively, in TTB1, while they read GTA and GTG, respectively, in TTB2. Likewise, the termination codons read TAG for the *cox*2, *nad*1, and *nad*5 genes in TTB1, while they read TAA for the same genes in the TTB2 genome. Finally, the length of the open reading frame (ORF) for some of the genes differed between the genomes. *nad*4 and *nad*4L showed different ORF lengths between TSDK and TSUG. For the TTB1 and TTB2 genomes, *nad*1, *nad*2, *nad*5, *nad*4, *nad*4L, *atp*6, and *atp*8 also varied in terms of respective ORF lengths. However, TTB1 and TTHUG were identical in terms of all initiation and termination codons and gene lengths, except for the *nad*4 gene, which was one amino acid (3 nucleotides) shorter in TTHUG.

### Comparative sequence analysis

Genetic distances between each PCG and rDNA gene of the different genomes of *Trichuris* spp. in primates and pigs are listed in Table 5, together with differences in amino acid sequences, based on all encoded proteins. The genetic distances between worms for individual PCGs and rDNA genes are given in S2 Table. The highest genetic variation was found in the *atp*8 gene, and the most conserved gene was *rrn*S. Among all the PCGs, *cox*1 and *atp*8 were found to be the most and least conserved gene, respectively. The overall differences in nucleotide and amino acid sequences between the genomes of TTHCH and TTHUG were high (18.8% and 14.6%, respectively), whereas the baboon *Trichuris* TTB1 was genetically nearly identical to the human TTHUG. TTB2 was most closely related to TTHCH with an overall nucleotide difference of 6.5%. Among the primate-derived *Trichuris*, T.GHL from francois' leaf-monkey was most distinct, with a nucleotide difference of 27%–28% compared with worms from humans and baboons. Nucleotide differences between TSUG and TSCH (3.1%) were much lower compared with TSDK (~9%).

**Table 5:** Overall genetic and protein distances between the *Trichuris* spp. genomes derived from baboons (TTB1 and TTB2), humans (TTHCH and TTHUG from China and Uganda, respectively), pigs (TSCH, TSUG and TSDK from China, Uganda and Denmark, respectively), and francois' leaf monkey (T.GHL). The amino acid sequence distances are given above the diagonal and genetic distances below the diagonal.

*Trichuris* genomes	TTHCH	TTHUG	TTB1	TTB2	T.GHL	TSDK	TSUG	TSCH
TTHCH		14.6	14.8	4.7	27.6	39.2	33.6	35.3
TTHUG	18.8		0.8	14.1	26.7	39.1	33.3	35.0
TTB1	19.9	1.0		14.4	26.7	39.1	33.2	35.0
TTB2	6.5	19.0	19.4		27.9	38.9	33.4	35.0
T.GHL	28.1	27.6	27.9	28.8		40.9	35.7	37.2
TSDK	31.2	30.3	30.6	31.6	32.0		5.5	6.1
TSUG	31.2	30.2	30.5	31.6	32.2	9.1		2.2
TSCH	30.9	30.4	30.7	31.1	32.2	8.8	3.1	

**Fig 1:**
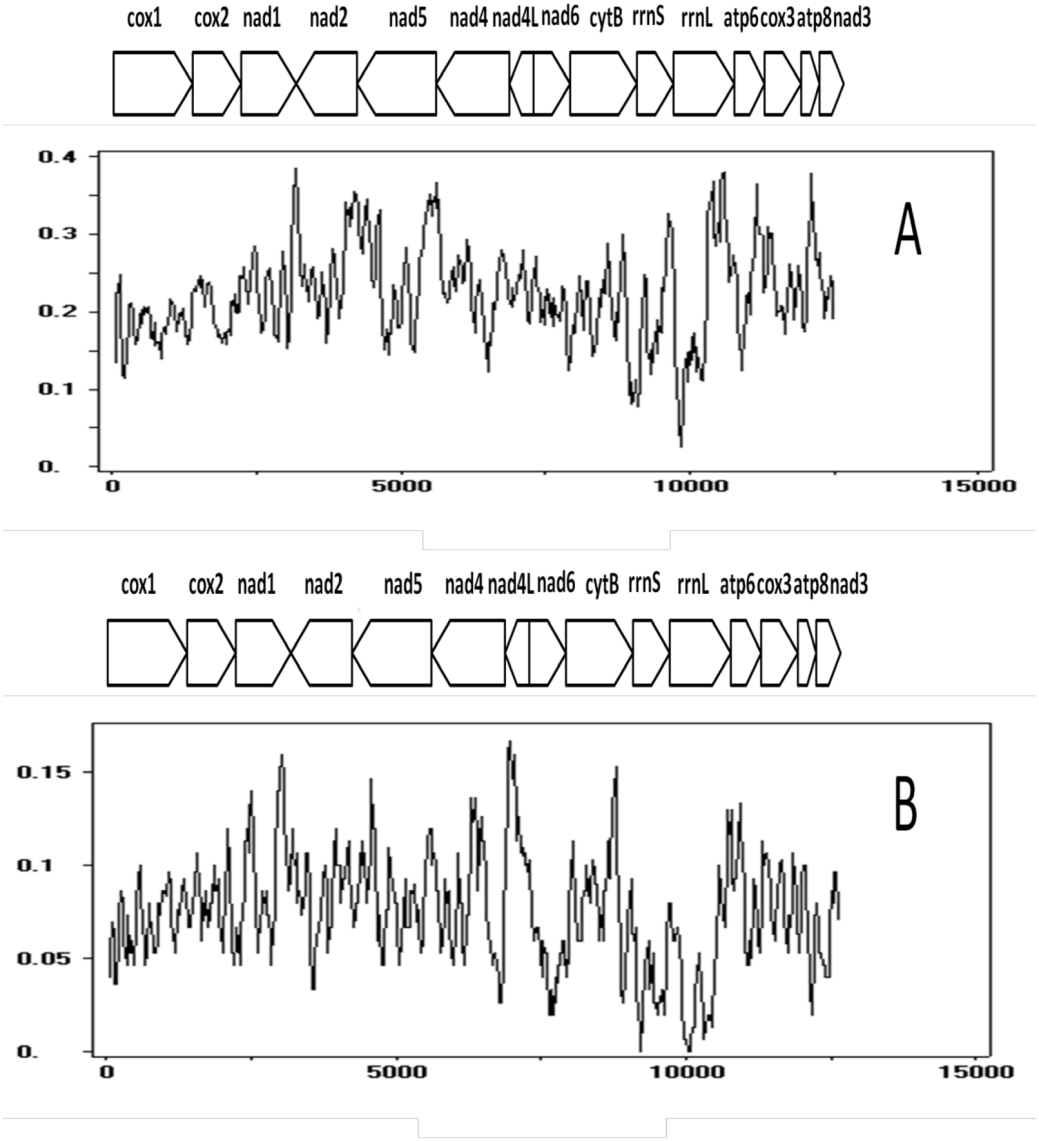
Nucleotide diversity (π) for protein coding regions and ribosomal DNA (*rrn*S and *rrn*L) measured every 25 bp over 100 bp windows. The aligned dataset for *Trichuris* in primates (humans, baboons, francois’ leaf monkey) is given in (A), while that of the pig-derived *Trichuris* is given in (B).

Nucleotide diversity among the *Trichuris* genomes was analyzed using the sliding window approach for all the PCGs and rDNA genes. The variation estimated for all primate- and pig-derived *Trichuris* is given in two separate windows (Fig 1). The overall variation within the primate-derived *Trichuris* was higher compared with that of pig-derived *Trichuris*. The rDNA and *cox*1 genes were found to have the lowest nucleotide diversity among pig- and primate-derived *Trichuris*.

### Phylogenetic analysis

**Fig 2:**
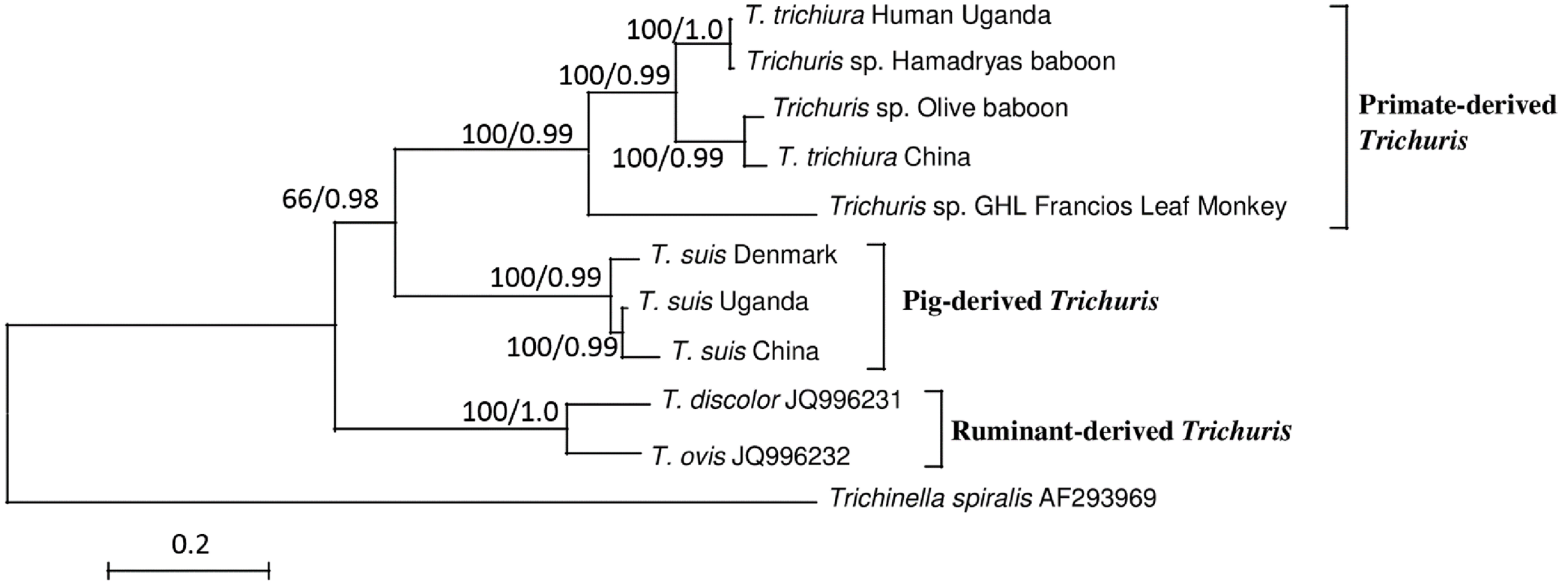
Inferred phylogenetic relationship among *Trichuris* spp. using concatenated amino acid sequences and Maximum Likelihood (ML) and Bayesian Inferences (BI). The three major groups identified by the phylogenetic tree include primate-, pig- and ruminant- derived *Trichuris*. Bootstrap frequencies (BF) and posterior probabilities (PP) are indicated on the branches (BF/PP). Scale bar represents the number of substitutions per site.

Amino acid and nucleotide data sets gave similar tree topologies by all the methods applied (NJ, ML and BI). The best-to-fit model was mtREV+G+I+F for the amino acid sequences and General Time Reversible with gamma distribution and invariant sites (GTR+G+I) for the nucleotide sequences. Three major groups were identified in the phylogeny based on the mt genomes (amino acid sequences), namely primate-, pig-, and ruminant-derived *Trichuris* (Fig 2). The nucleotide sequence-based phylogeny is provided in S1 Fig and depicts similar tree topology.

### *Cox*1 phylogeny

**Fig 3:**
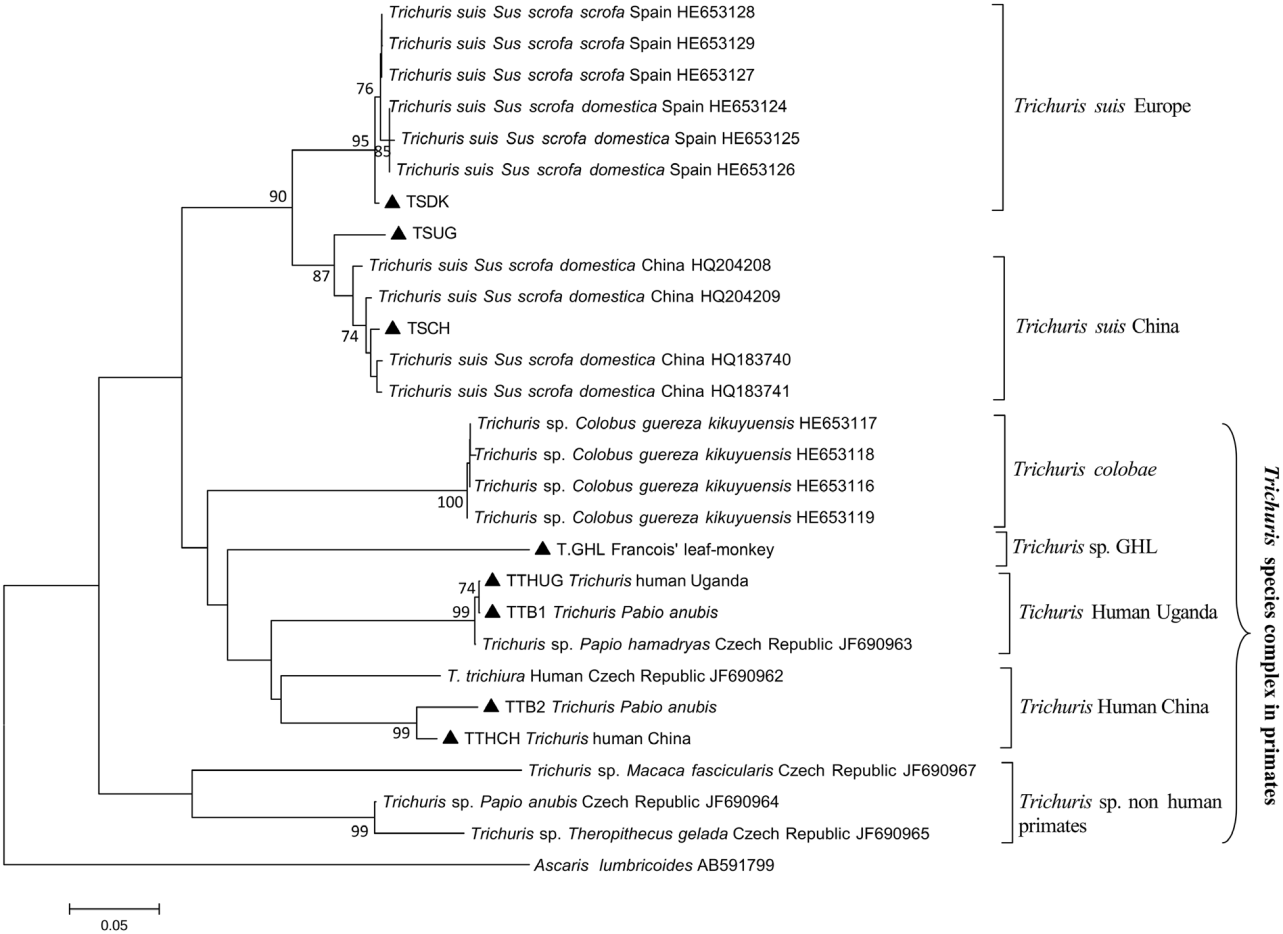
Inferred phylogeny among *Trichuris* spp. recovered from pigs and primates based on partial *cox*1 sequences and NJ clustering. Samples for which the full mitochondrial genome is sequenced are indicated by a solid triangle. The phylogeny identified two major clades for *Trichuris* in pigs, namely ‘*T*. *suis* Europe’ and ‘*T*. *suis* China’. Five distinct clades for *Trichuris* spp. recovered from primates (including humans) were identified, namely ‘*T*. *colobae*’, ‘*Trichuris* sp. GHL’, ‘*Trichuris* Human Uganda’, ‘*Trichuris* Human China’, and ‘*Trichuris* sp. non-human primates’. Scale bar indicates number nucleotide substitutions per site. Only bootstrap values > 70 are given.

For the *cox*1 sequences, the best data fit was obtained with the Tamura 3-parameter model with gamma distribution. Both NJ and ML depicted similar tree topologies; hence, the NJ tree is depicted in Fig 3. TSDK clustered with Spanish *T*. *suis* and forms the '*T*. *suis* Europe' clade, and TSCH is found in the *'T*. *suis* China' clade. TSUG is most closely related to *T*. *suis* from China, which is concordant with the mt genome phylogeny. The *cox*1 phylogeny also supports the presence of a *Trichuris* species complex infecting primates, identifying five distinct clades, which were named after the *Trichuris* spp. recovered from these hosts, namely *T*. *colobae* recovered from *Colobus guereza kikuyensis* [29] and *Trichuris* sp. GHL from francois' leaf monkey [7]. Human-derived *Trichuris* TTHUG and TTHCH were found in two separate clades named after the country of origin, 'Human *Trichuris* Uganda' and 'Human *Trichuris* China', respectively, while the last clade comprises *Trichuris* sp. from different non-human primates (*Theropithecus gelada*, *Macaca fascicularis*, and *P*. *anubis*) and here named '*Trichuris* sp. non-human primates'. The baboon *Trichuris* TTB1 clusters with the *Trichuris* from baboon (*P*. *hamadryas*) in the 'Human *Trichuris* Uganda' clade, while TTB2 clusters with the 'Human *Trichuris* China' according to the mt genome phylogeny (Fig 2). The human *Trichuris* from Czech Republic is found clustering with the human *Trichuris* from China. Remarkably, *Trichuris* sp. from *P*. *anubis* from the Czech Republic is genetically very distinct and clusters in a clade (*Trichuris* sp. non-human primates) distant to that of TTB2, although both are *Trichuris* isolated from the same host species.

## Discussion

We sequenced the complete mt genomes of *Trichuris* spp. recovered from a human, baboons, and pigs and evaluated their genetic and evolutionary relationships. Several major haplotypes with clear genetic distinctiveness were observed, suggesting that multiple *Trichuris* species infect these host species and supporting the hypothesis that whipworms in primates comprise a species complex, which may also be the case for whipworms in pigs (S3 Fig).

The two human *Trichuris* from Uganda and China were genetically distinct, and the difference in amino acid and nucleotide sequences was found to be around 14.6% and 18.8%, which is in the range of previously reported differences between different parasitic nematode species, suggesting the presence of at least two *Trichuris* species infecting humans. For instance, the difference in amino acids for mt protein sequences between *T*. *ovis* and *T*. *discolor* adds up to 15.4% [28], 11.7% between *Wuchereria bancrofti* and *Brugia malayi* [30], 10.3% between *Chabertia ovina* and *C*. *erschowi* [31], and ranges from 4% to 18% between different species of *Trichinella* [32]. The baboon *Trichuris*, TTB1, was nearly identical to the human *Trichuris* from Uganda, which is in accordance with a previous study analyzing beta tubulin genes and the ITS-2 region [6], while the other baboon *Trichuris*, TTB2, was genetically more related to the human *Trichuris* from China, suggesting that baboons—similar to humans—may also host at least two *Trichuris* spp. In accordance with our study, but based on ITS-1 and -2 sequence analyses, Ravasi et al. [5] identified two different *Trichuris* species in humans from Cameroon and China, which were also found in chacma baboons in South Africa. On the other hand, *Trichuris* from the leaf monkey was very distinct from baboon worms, suggesting different *Trichuris* species in non-human primates as proposed by Liu et al. [7]. Indeed, Ghai et al. [8] recently suggested that primates may be infected with several *Trichuris* species, with some species only found in humans and others only found in non-human primates, while others again are shared, suggesting various degrees of host specificity of the different *Trichuris* spp. in primates.

Amino acid sequence distances between TSDK compared with TSCH and TSUG were considerable (around 6.1% and 5.5%, respectively), while TSCH and TSUG were genetically more closely related (2.2%). Although the distances between TSDK, TSUG, and TSCH were not notably high, similar amino acid sequence distances between different parasitic nematode species have been reported, such as bewteen *Ancylostoma duodenale* and *A*. *caninum* (4%) [33,34] and different *Toxocara* spp. (5.6%–7.2%) [35], suggesting that pigs may also harbor different *Trichuris* species.

The *cox*1 phylogeny also supports that whipworms in primates and pigs make up a cryptic species complex. The human *Trichuris* TTHCH and TTHUG cluster in two distinct clades, here designated 'Human *Trichuris* China' and 'Human *Trichuris* Uganda'. The previously described *Trichuris* species from different non-human primates (*T*. *colobae* and *Trichuris* sp. GHL in black-and-white colobus and francois' leaf monkey, respectively) [7,29] were also found in distinct clades (Fig 2). Moreover, one of the clades included whipworms from other non-human primates (olive, gelada baboons, and long-tailed macaque), which could represent a different *Trichuris* species in non-human primates (‘*Trichuris* sp. non-human primates’ clade). Hence, the *cox*1 phylogeny suggested at least five potential *Trichuris* spp. infecting primates. Likewise, Ghai et al. [8] identified a distinct group of worms found only in non-human primates, and these might be related to the ‘*Trichuris* sp. non-human primates’ clade in our study. However, the *Trichuris* from a black-and-white colobus was not identified as a separate species by Ghai et al. [8], suggesting that this host can also be infected with different *Trichuris* spp., or it may reflect the use of different genetic markers between studies [36]. For pig *Trichuris*, the *cox*1 phylogeny identified the *T*. *suis* from Spain to be genetically closely related to TSDK (‘*T*. *suis* Europe’ clade) but distinct from *T*. *suis* from China, supporting the possibility that different *T*. *suis* species can be found in various geographical regions.

In addition to obvious transmission issues for *Trichuris* species that are shared between humans and non-human primates, the presence of different cryptic species might also be very important for implementation of appropriate control strategies. For instance, different cryptic species of the human trematode *Opisthorchis viverrini* in different localities (Laos and Thailand) were found to have significantly different fecundity as measured by eggs/g/worm [37]. Moreover, benzimidazole resistance has been associated with single nucleotide polymorphisms (SNPs) in the beta tubulin gene and has been detected in *T*. *trichiura* [38], but the presence and frequencies of these SNPs may vary with geography [6, 38] and between whipworms within the species complex. Hence, control and treatment in different areas may not be equally effective, and therefore, there is a need to further explore the species diversity and compare the pathology, epidemiology, and drug susceptibility of different *Trichuris* species [13].

In conclusion, based on complete mt genome analyses, we suggest the existence of a *Trichuris* species complex in primates and pigs. Moreover, a rich source of genetic markers is provided that can be used to inform further investigation into the genetic variation among *Trichuris* spp. infecting these hosts. There is an urgent need to further elucidate the *Trichuris* species infecting primates in order to illuminate transmission routes and to identify and implement appropriate control measures. Consequently, differences in pathology and treatment efficacy between species should be investigated. This study also suggests that *Trichuris* in pigs may consist of a cryptic species complex with similar implications. However, this hypothesis needs further testing including samples from various geographical regions and including nuclear DNA markers as well.

## Supporting Information

S1 Table Summary of the 15 primers used for amplification of the mitochondrial genome of the human Trichuris sample, TTHUG. (XLSX)

S2 Table Pairwise genetic and protein distances for the different mitochondrial protein-coding and rDNA (*rrn*S and *rrn*L) genes for different *Trichuris* in different hosts in different countries, including worms from humans in Uganda (TTHUG) and China (TTHCH); baboons (TTB1 and TTB2); pigs from Uganda (TSUG), China (TSCH), Denmark (TSDK) and francois’ leaf monkey (T.GHL). (XLSX)

S1 Fig Inferred phylogenetic relationship among *Trichuris* spp. using concatenated nucleotide sequences of protein-coding genes and the Maximum Likelihood (ML). Bayesian Inferences revealed a similar tree topology. Bootstrap frequencies (BF) and posterior probabilities (PP) are indicated on the branches (BF/PP). Scale bar represents the number of nucleotide substitutions per site.
(JPG)
