# Supplementary figures and images for: Mitochondrial Genome Analyses Suggest Multiple Trichuris Species in Humans, Baboons, and Pigs from Different Geographical Regions
Source: PLoS Negl Trop Dis. 2015 Sep 14;9(9):e0004059. doi: 10.1371/journal.pntd.0004059 (PMC4569395; doi:10.1371/journal.pntd.0004059)

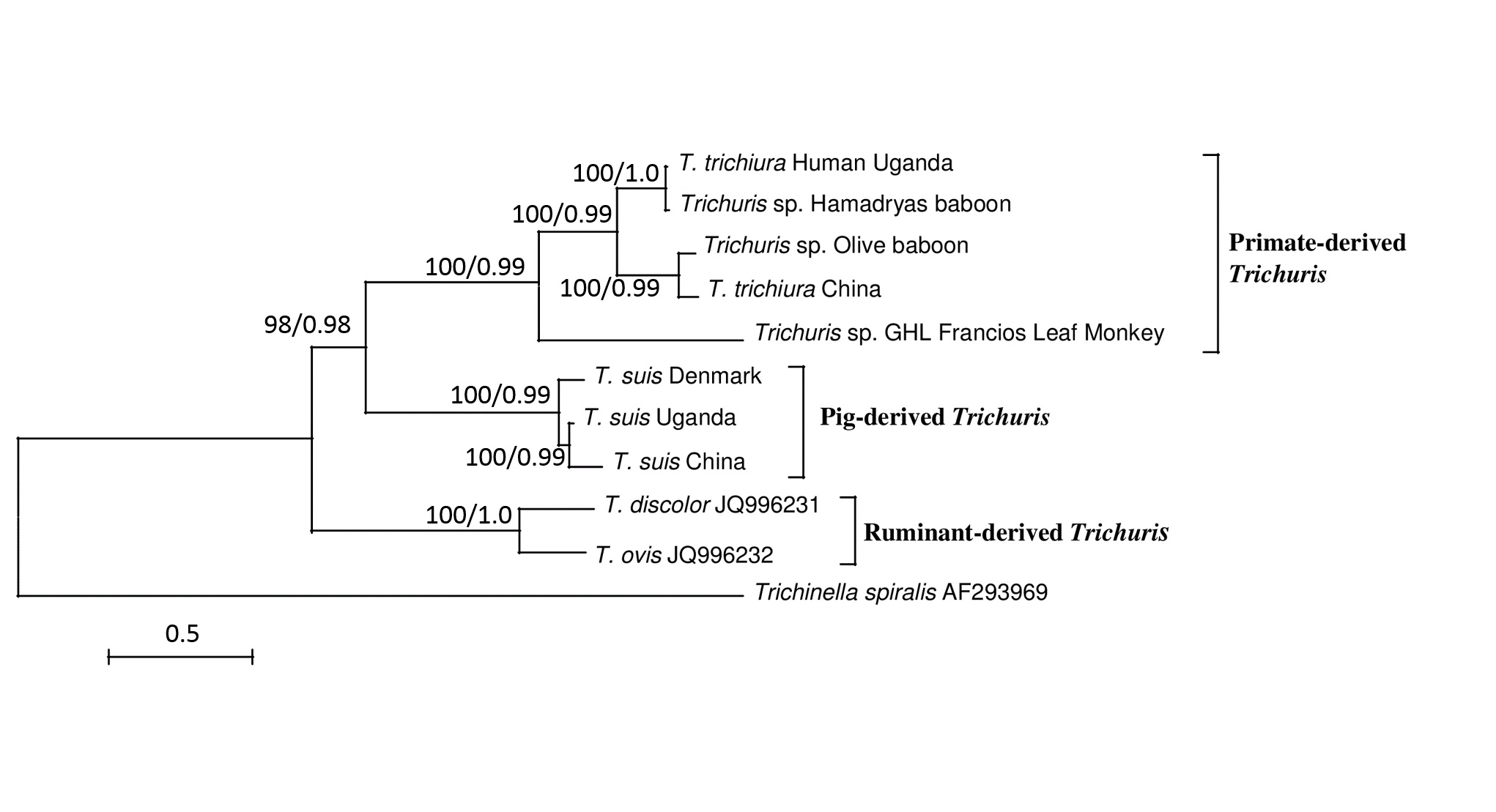

Supplement: S1 Fig — Bayesian Inferences revealed a similar tree topology. Bootstrap frequencies (BF) and posterior probabilities (PP) are indicated on the branches (BF/PP). Scale bar represents the number of nucleotide substitutions per site. (JPG) [file pntd.0004059.s003.jpg]
